# The Critical Role of the Tumor Microenvironment in Shaping Natural Killer Cell-Mediated Anti-Tumor Immunity

**DOI:** 10.3389/fimmu.2013.00490

**Published:** 2013-12-25

**Authors:** Joanna Baginska, Elodie Viry, Jérôme Paggetti, Sandrine Medves, Guy Berchem, Etienne Moussay, Bassam Janji

**Affiliations:** ^1^Laboratory of Experimental Hemato-Oncology, Department of Oncology, Public Research Center for Health (CRP-Santé), Luxembourg City, Luxembourg

**Keywords:** hypoxia, natural killer cells, autophagy, tumor-derived exosomes, tumor microenvironment

## Abstract

Considerable evidence has been gathered over the last 10 years showing that the tumor microenvironment (TME) is not simply a passive recipient of immune cells, but an active participant in the establishment of immunosuppressive conditions. It is now well documented that hypoxia, within the TME, affects the functions of immune effectors including natural killer (NK) cells by multiple overlapping mechanisms. Indeed, each cell in the TME, irrespective of its transformation status, has the capacity to adapt to the hostile TME and produce immune modulatory signals or mediators affecting the function of immune cells either directly or through the stimulation of other cells present in the tumor site. This observation has led to intense research efforts focused mainly on tumor-derived factors. Notably, it has become increasingly clear that tumor cells secrete a number of environmental factors such as cytokines, growth factors, exosomes, and microRNAs impacting the immune cell response. Moreover, tumor cells in hostile microenvironments may activate their own intrinsic resistance mechanisms, such as autophagy, to escape the effective immune response. Such adaptive mechanisms may also include the ability of tumor cells to modify their metabolism and release several metabolites to impair the function of immune cells. In this review, we summarize the different mechanisms involved in the TME that affect the anti-tumor immune function of NK cells.

## Introduction

Natural killer (NK) cells are potent cytolytic lymphocytes belonging to the innate immune system. NK cells comprise up to 15% of all circulating lymphocytes and are also found in peripheral tissues including the liver, peritoneal cavity, and the placenta. Although resting NK cells circulate in the blood, they are capable of infiltrating most cancer tissues following activation by cytokines. NK cells can be rapidly activated in the periphery by NK cell stimulatory factors, such as interleukin (IL)-12, interferon (IFN)-α and -β, IL-15, or IL-2 (1). Regulation of NK cell activity depends on the repertoire of germline-encoded activating and inhibitory receptors. The activating receptors recognize stress-induced, pathogen-derived, or tumor-specific ligands, whereas the inhibitory receptors bind self-molecules presented on normal cells. Owing to a diversified set of inhibitory and activating receptors, NK cells are capable of recognizing and killing an array of tumor cells (2). Beyond innate activity, NK cells are important for the regulation of anti-tumor adaptive immunity (3, 4).

In addition to their well-described role in inhibiting the early stage of tumor formation, NK cells are able to eradicate large solid tumors. Such eradication depends on the massive infiltration of proliferating NK cells due to the release and the presentation of IL-15 by cancer cells in the tumor microenvironment (TME). It has been shown that infiltrating NK cells are strikingly similar morphologically to uterine NK cells (5).

Based on the fact that NK cells can eliminate cancer cells in experimental conditions, it has been proposed that NK cells can be used clinically in therapeutic settings against cancer. Importantly, data from haploidentical hematopoietic stem cell transplantation and NK cell-based adoptive immunotherapy support the clinical effects of NK cells (6). Based on our current knowledge of the molecular specificities that regulate NK cell functions, it is tempting to speculate that a design of tailored NK cell-based immunotherapeutic strategies against cancer might be possible.

Recent data confirm that NK cells are required for the induction of potent anti-tumor-specific cytotoxic T lymphocytes (T cells) responses, by a mechanism involving dendritic cell (DC) editing (7, 8). Furthermore, NK cells can recognize tumors that might evade T cell-mediated killing by aberrant human leukocyte antigen (HLA) expression (9), indicating that NK cells participate in tumor immunosurveillance.

A significant correlation between high intratumoral levels of NK cells and increased survival has been shown in several types of cancer (10). Indeed, high levels of NK-infiltrating tumors have been associated with a significant improvement of clinical outcomes in patients with head and neck squamous carcinoma (HNSCC). It has been reported by van Herpen et al. that CD56+ NK cells in lymph nodes produced considerable amounts of IFN-γ that subsequently lead to tumor regression in IL-12-treated HNSCC patients (11). A direct positive correlation between the density of CD57+ NK cells and a good prognosis has been reported for oral squamous carcinoma (12) and gastric carcinoma (GC) tumors (13). In addition, NK cell infiltration was found to also correlate with the depth of invasion, the clinical stage, and the venous invasion. Therefore, the 5-year survival rate of GC patients with a high rate of NK infiltration was significantly better than that of patients with a low level of NK infiltration (13).

Natural killer-based immunotherapy is a promising strategy for solid and hematologic cancers and it can potentially be combined with chemotherapy, radiation, or monoclonal antibody therapy. For example, the proteasome inhibitor bortezomib (Velcade^®^), which is clinically approved for the treatment of refractory/relapsed myeloma, downregulates the expression of major histocompatibility complex (MHC) class I on the target cell surface and thereby shifts the balance toward NK cell activation and target cell killing (14). Therefore, such combination therapy has important therapeutic implications for multiple myeloma (MM) and NK cell-related malignancies in the context of adoptively transferred allogeneic and autologous NK cells (15). NK cell-based therapy can be combined with radiation therapy as irradiation-induced tissue injury increases the expression of NK-activating ligands (e.g., NKG2D ligands) on malignant cells, thereby rendering tumors more susceptible to NK cell cytotoxic activity (16) Another NK cell-based approach used in therapy is the antibody-dependent cellular cytotoxicity (ADCC). This approach is based on the ability of NK cells, expressing an activating Fc receptor, to kill tumor cells by recognizing the constant region of tumor-bound monoclonal antibodies (mAbs). Clinically, ADCC strategy has been used in CD20+ lymphoma patients treated with rituximab (Rituxan™) (17) or HER2/neu-expressing breast cancer patients treated with trastuzumab (Herceptin™) (18). It is important to note that the co-administration of immunomodulatory cytokines (e.g., IL-12) can enhance the effects of anti-tumor mAbs via the activation of NK cells *in vitro*. This effect has been observed in breast cancer patients overexpressing HER2/neu and treated with IL-12 and trastuzumab in a phase I trial (19).

**Figure 1:**
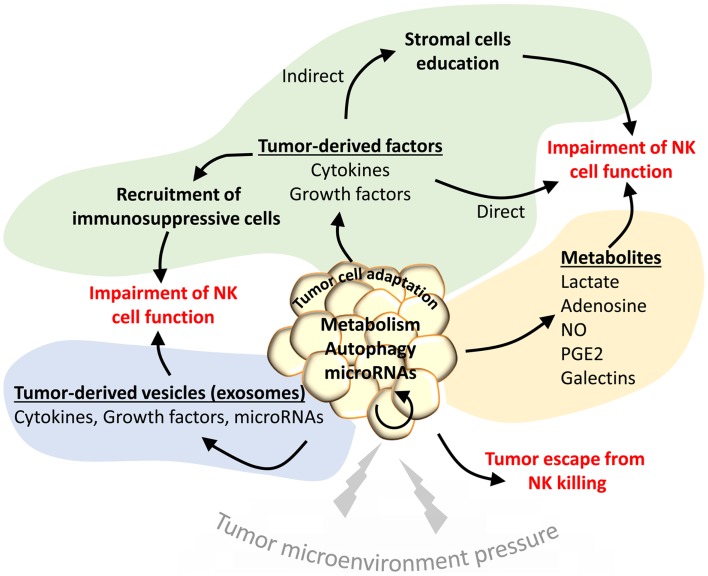
**The tumor microenvironment activates different mechanisms to impair the NK-mediated anti-tumor immunity**. Under the pressure of the tumor microenvironment (TME), tumor cells adapt to such stress by activating intrinsic resistance mechanisms (autophagy) or by regulating their metabolism. Such regulation leads to the secretion of several metabolites that impair the function of NK cells in the tumor site (yellow area). Tumor cells under stress conditions may activate the release of tumor-derived vesicles containing cytokines, growth factors, or microRNAs to directly impact the NK functions (blue area). Such factors can be secreted directly in the TME to recruit immunosuppressive cells or to educate stromal cells involved in the impairment of NK cell functions (green area).

Despite the progress made in the field of NK-based immunotherapy, there are still many obstacles to eliciting an effective immune response. One major impediment is the ability of tumor cells to activate several mechanisms that lead to tumor escape from NK-mediated killing. It has become increasingly clear that the TME plays a crucial role in the impairment of the immune response and in the development of many overlapping mechanisms that create an immunosuppressive microenvironment. It has been reported that tumor-associated NK cells display a modified phenotype, thereby supporting the notion that tumor-induced alterations of activating NK cell receptor expression may hamper immune surveillance and promote tumor progression (20). Decreased cytotoxic activity of NK cells infiltrating tumors was also observed in different types of human cancer such as lung carcinoma (21), indicating that the TME is a critical factor influencing NK-mediated killing of tumor cells. Hypoxia, a characteristic feature of advanced solid tumors resulting from defective vascularization and a subsequent insufficient oxygen supply, is considered one of the hallmarks of the TME (22). It is now well established that hypoxia contributes to malignant progression in cancer by inducing an invasive and metastatic phenotype of tumor cells and by activating resistance mechanisms to different anti-cancer therapies (23). Extensive efforts have been made in recent years to identify these mechanisms. We review here how the local microenvironment, in the particular context of hypoxia, impacts NK cell responsiveness and shapes the anti-tumor response (Figure 1).

## Tumor-Derived Factors Create an Immunosuppressive Microenvironment for NK Cell Functions

### Impairment of NK cell function by cells from the tumor microenvironment

Hypoxic tumor cells have the ability to activate resistance mechanisms to create an immunosuppressive microenvironment. Indeed, through their ability to produce cytokines such as tumor necrosis factor (TNF)-α and stromal cell-derived factor 1 (SDF-1), hypoxic tumor cells induce the homing of bone marrow-derived CD45+ myeloid cells to tumor areas (24). The invasion of myeloid cells in the TME is reported to be a highly immunosuppressive factor for NK cells (25). Myeloid-derived suppressor cells (MDSCs) are one of the major components of the immune-suppressive network responsible for the impairment of NK cell- and T cell-dependent anti-cancer immunity (26). The immunosuppressive function of MDSCs is related to their production of IL-10 that decreases the production by macrophages of IL-12, a pro-inflammatory cytokine involved in the activation of NK cells (27). It has also been shown that cancer-expanded MDSCs induce anergy of NK cells by inhibiting cytotoxicity, NKG2D expression, and IFN-γ production through membrane-bound transforming growth factor (TGF)-β (28). Furthermore, it has been demonstrated that hypoxia, via the induction of hypoxia-inducible factor (HIF) 1-α in MDSCs, is responsible for their differentiation to tumor-associated macrophages (TAMs) (29). Although macrophages contribute to tumor cell death in the early immune response to neoplasia, their presence in the TME correlates with a poor prognosis for patients with advanced stages of cancer (30, 31).

**Figure 2:**
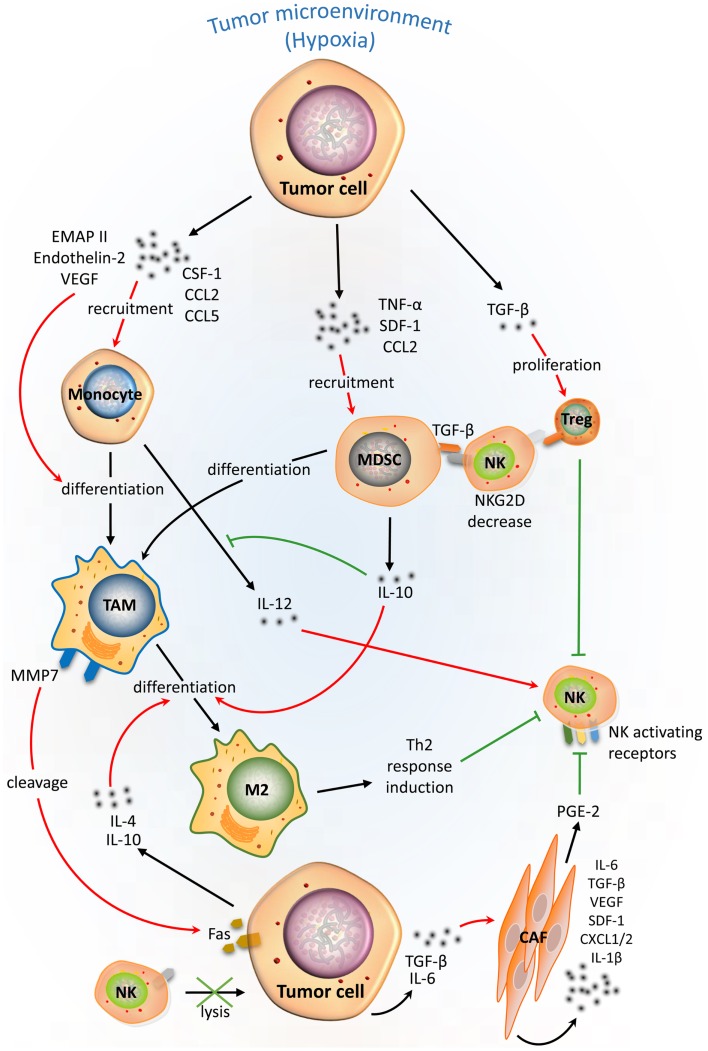
**Complex cellular interplay within the hypoxic tumor microenvironment inhibits NK-mediated killing**. Tumor cells in a hypoxic tumor microenvironment (TME) secrete soluble factors that educate immune cells [e.g., monocytes, tumor-associated macrophages (TAMs), myeloid-derived suppressor cells (MDSCs), and regulatory T cells (Treg)], and stromal cells such as cancer-associated fibroblasts (CAFs). This scheme summarizes the effects of tumor-derived soluble factors on recruitment, differentiation, proliferation, and activation of tumor-associated cells (red arrows) in the hypoxic TME and their immunosuppressive activities (green lines) on NK-mediated lysis of tumor cells.

Macrophages constitute another major myeloid component of the infiltrated tumors and can comprise up to 80% of the cell mass in breast carcinoma (32). Hypoxic tumor secrete chemoattractants [e.g., colony-stimulating factor (CSF)-1, CC chemokine ligands (CCL) 2 and 5], resulting in the recruitment of monocytes from the blood to the tumor site. Infiltrated monocytes differentiate into CD206+ TAMs and accumulate in hypoxic areas of endometrial, breast, prostate, and ovarian cancers (30). This process is driven by tumor-secreted molecules such as endothelial monocyte-activating polypeptide (EMAP) II, endothelin 2, and vascular endothelial growth factor (VEGF) and also by the inhibition of the CC chemokine receptors (CCRs) 5 and 2 expression (33). Exposure of TAMs to tumor-derived cytokines such as IL-4 and IL-10 converts the TAMs into polarized type II or M2 macrophages owing to the immunosuppressive and pro-angiogenic activities. Subsequently, M2 macrophages establish an environment that skews CD4+ and CD8+ T cell immunity toward a tumor-promoting type 2 response (34). It has been also demonstrated that hypoxia upregulates the expression of the matrix metalloproteinase (MMP)-7 protein on the TAM surface, leading to the cleavage of Fas ligand from neighboring cancer cells, making them less responsive to NK cells and T cell-mediated lysis (35) (Figure 2).

Recently, a link between tumor hypoxia and immune tolerance to NK cells through the recruitment of regulatory T (Treg) cells has been established. Hypoxia induces secretion of the immunosuppressive cytokine TGF-β from gastric cancer cells, which subsequently induces the proliferation and the accumulation of Treg cells in the TME (36). Moreover, human Treg cells induce anergy of NK cells through membrane-bound TGF-β and subsequently downregulate the activating receptor NKG2D on the surface of NK cells (37).

The immunosuppressive microenvironment can also be created through the ability of cancer cells to activate cancer-associated fibroblasts (CAFs) via the release of TGF-β or IL-6 (38, 39). CAFs have been shown to sharply interfere with NK cells cytotoxicity and cytokine production. Notably, it has been reported that CAFs are able to inhibit the IL-2-induced upregulation of the activating receptors NKp44, NKp30, and DNAX accessory molecule-1 (DNAM-1) at the NK cell surface. NKp44 and NKp30 expression is modulated by prostaglandin E2 (PGE2) released from CAFs, while DNAM-1 regulation requires cell-to-cell interaction. Such inhibition results in impaired NK cell-mediated killing of melanoma target cells (40). Likewise, CAFs directly impact cells of the TME and/or attract additional cells to the tumor site by secreting numerous factors including IL-6, TGF-β, VEGF, SDF-1, CXCL1/2, and IL-1β (41) (Figure 2).

Other mechanisms implicated in the establishment of immune-suppressive microenvironment are the expression of the immune checkpoint receptors, cytotoxic T-lymphocyte antigen (CTLA)-4, and the programed death receptor (PD)-1. Such receptors appear to play important roles in anti-tumor immunity and have been most actively studied in the context of clinical cancer immunotherapy. However, the effect of the TME on their regulation is poorly investigated. Nevertheless, the TME has been shown to mediate the induction of the PD-1 pathway (42). In line with this observation, NK cells from MM patients express PD-1, whereas normal NK cells do not. Anti-PD-1 antibody-based therapy enhances human NK cell function against autologous primary MM cells (43), highlighting the role of the PD-1/PD-L1 signaling axis in NK-mediated immune response against tumors. There is no direct evidence so far linking hypoxia and the induction of CTLA-4 expression and the PD-1/PD-L1 pathway. Further investigations are required to determine the precise role of the TME in the regulation of CTLA-4 and the PD-1/PD-L1 pathways.

### Inhibition of NK cells by tumor cell-derived factors

**Figure 3:**
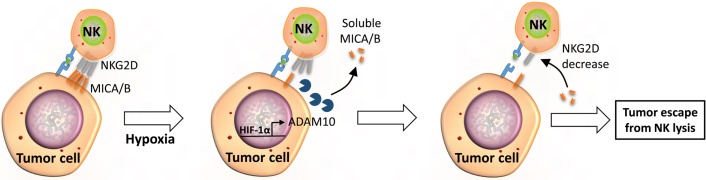
**Soluble MICA/B regulate NKG2D receptors on the surface of NK cells**. Under hypoxic stress, tumor cells activate expression through HIF-1α and the release of ADAM10. Released ADAM10 cleaves MICA/B ligands on the surface of tumor cells and soluble MICA/B downregulates the expression of NKG2D on the surface of NK cells, leading to tumor escape from NK-mediated killing.

The MHC class I chain-related (MIC) molecules, MICA and MICB, as well as the UL16-binding proteins (ULBPs), expressed on the surface of a broad range of carcinomas and some hematopoietic malignancies, play an important role in tumor surveillance by NK cells. The interaction of cell surface MIC molecules with NKG2D receptors on NK cells is critical to activate target cell killing. In this context, hypoxia has been reported to increase MICA shedding from the surface of cancer cells through the impairment of nitric oxide (NO) signaling and therefore affect the NK-mediated killing of target cells. Soluble MIC leads to a downregulated expression of NKG2D and CXC chemokine receptor (CXCR) 1 on the NK cell surface (44). This mechanism involves the HIF-1α-dependent upregulation of A disintegrin and metalloproteinase domain-containing protein (ADAM) 10, which subsequently decreases the level of MICA on the tumor cell membrane (44, 45) (Figure 3).

In addition, hypoxic stress can induce the formation of dimers of the non-classical MHC class I molecule HLA-G at the surface of melanoma cells, thereby protecting tumor cells from NK-mediated killing. It appears that such induction is mediated by secretion of IFN-β and -γ and by direct interaction of HLA-G with NK cells (46).

Inhibiting the expression of activating NK cell receptors, including NKp30, NKp44, and NKG2D, has been shown to impair NK cell-mediated cytolytic activity in a model of melanoma (47). Although NK cells in the TME adapt and survive hypoxic stress by upregulating HIF-1α, they lose the ability to upregulate the surface expression of NKp46, NKp30, NKp44, and NKG2D receptors in response to IL-2 or other activating cytokines (e.g., IL-15, IL-12, and IL-21). However, it is important to note that hypoxia does not significantly alter the surface density and the function of the Fc-γ receptor CD16, thus allowing NK cells to maintain their capability of killing target cells via ADCC (48).

In addition to solid tumors, the immunosuppressive effect of the hypoxic TME has been also described in MM cells as hypoxia reduced NK cell killing of MM cell lines despite an unchanged NK cell degranulation level. In addition, hypoxia did not alter the surface expression of NK cell ligands (HLA-ABC and -E, MICA/B, and ULBP1-2) and receptors [killer cell Ig-like receptors (KIR), NKG2A/C, DNAM-1, natural cytotoxicity receptors (NCR), and 2B4], but decreased the expression of the activating NKG2D receptor and intracellular level of perforin and granzyme B. Pre-activation of NK cells by IL-2 removed the detrimental effects of hypoxia and increased NKG2D expression (49).

It is now well documented that the killing capacity of NK cells can be potentiated by cytokines such as IFN-γ and IL-2 (50, 51). Besides its effect of damping the cytotoxic activity of NK cells, hypoxia substantially decreases the ability of NK cells to be activated by IFN-γ through a mechanism that is not fully understood (52). Overall, it appears that manipulation of the TME will be an important consideration in achieving optimal NK-mediated, anti-tumor responses.

Since NKG2D ligand mRNAs are expressed in normal tissues, it has been proposed that their expression might be regulated at the post-transcriptional level by microRNAs (miRNAs) (53). Indeed, a subset of endogenous cellular miRNAs is proposed to repress MICA and MICB by targeting their 3′ UTR regions (54). Upon stress induction, the increase in MICA and MICB transcription might exceed the inhibitory function of miRNAs, whose expression remains constant, and result in an overexpression of MICA and MICB. Interestingly, among this subset of miRNAs, miR-17-5p, miR-20a, miR-93, miR-106b, miR-372, miR-373, and miR-520 have been shown to be overexpressed in various tumors and be involved in tumor progression and invasion. Therefore, a new function of these miRNAs has been proposed in the impairment of the immune response through the regulation of MICA and MICB expression (Figure 1). Based on these observations, a “miRNA-based immunoevasion” model has been described that highlights intracellular cancer-associated miRNAs as important factors able to impair immune recognition through the targeting of NK ligands (54). Furthermore, miR-10b, an important “metastamir,” has been described to downregulate MICB and decrease the NKG2D-dependent cytotoxicity of NK cells (55). MiR-520b, an IFN-γ-induced miRNA, has been described to regulate MICA expression at both the transcriptional and post-transcriptional levels (56). It has also been proposed that viruses can take advantage of miRNA-based immunoevasion. Indeed, the hcmv-miR-UL112 encoded by the human cytomegalovirus impairs NK cell function during viral infection through the modulation of MICB expression (57). In addition, hcmv-miR-UL112 acts synergistically with the cellular miR-376a to induce escape from NK-mediated immune elimination (58). Together, these studies highlight the importance of miRNAs in the regulation of NKG2D ligand expression and tumor immune surveillance. Whether the expression of such miRNAs is regulated by hypoxia in the TME remains to be investigated.

## Tumor Microenvironment-Dependent Modulation of Cancer Cell Metabolism Affects NK Cell Functions

Through the sensing of oxygen level and/or the transcriptional activity of HIF-1α, hypoxia plays a key role in the reprograming of cancer cell metabolism. Indeed, reduced O_2_ availability induces HIF-1α, which regulates the transcription of a set of genes that encode proteins involved in various aspects of cancer biology (59). A well-known example is the shift of glucose and energy metabolism from oxidative to glycolytic metabolism that allows for the maintenance of redox homeostasis under conditions of prolonged hypoxia (60). The effects of such metabolic adaptations evolved by hypoxic cancer cells have received particular attention in the establishment of immune tolerance. In this section, we will focus on the mechanisms involved in tumor metabolism adaptation that participate in shaping the NK cell anti-tumor response within a hypoxic microenvironment (Figure 1).

### Lactate

To adapt to oxygen deprivation, hypoxic cancer cells undergo a dramatic alteration of cellular glucose metabolism characterized by a high glycolytic activity. HIF-1α plays a central role in this metabolic switch by inducing the expression of multiple genes involved in glucose uptake (glucose transporters-1 and -3) and metabolism (i.e., hexokinases-1 and -2 and lactate dehydrogenase A) (61). In addition, HIF-1α regulates the expression of monocarboxylate transporter 4 and pyruvate dehydrogenase kinase 1, thereby inhibiting the conversion of pyruvate to acetyl CoA (62). The accumulation of pyruvate in cells prevents its metabolism through the tricarboxylic acid cycle in mitochondria. Pyruvate is subsequently reduced to lactate and finally released from the tumor cells. It has been recently reported that cancer cells escape immune response through the release of lactate in the microenvironment and the presence of a low extracellular pH, as a consequence of the “Warburg effect” induced under hypoxia. *In vivo* and *in vitro* evidence has been provided indicating that tumor-derived lactate directly and indirectly alters NK cell functions. The direct effect involves the impairment of the cytolytic activity of NK cells by downregulating NKp46 expression and reducing perforin/granzyme B production. Moreover, lactate affects the NK-mediated killing indirectly through the increased MDSCs generation from mouse bone marrow, thus creating an immunosuppressive microenvironment. Interestingly, these immunosuppressive effects were efficiently reverted in a lactate dehydrogenase A-depleted cancer model (63).

### Adenosine

Hypoxia-driven accumulation of adenosine in the TME has been identified as another mechanism for immune modulation (64). It has been reported that the concentration of adenosine in the extracellular fluid of solid carcinomas may be increased up to 20-fold compared with normal tissues (65). The accumulation of adenosine is sustained, at least in part, by the hypoxia-mediated modulation of enzymes implicated in adenosine metabolism (i.e., adenosine kinase, endo-5′-nucleotidase). Moreover, the additional generation of extracellular adenosine from extracellular ATP occurs through the sequential enzymatic activity of the membrane-bound nucleotidases CD39 and CD73. It has been shown that CD73, involved in the dephosphorylation of AMP to adenosine, is upregulated by HIF-1α (66, 67). Once released in the extracellular environment, adenosine exerts various immunomodulatory effects via binding on adenosine receptors (i.e., A1, A2A, A2B, and A3) expressed on multiple immune subsets including NK cells.

In contrast to other immune cells such as macrophages and neutrophils, the effect of extracellular adenosine on NK cells is not fully known. Adenosine has been shown to inhibit TNF-α release from IL-2-stimulated NK cells and suppress their proliferation (68). Another study reported that adenosine inhibits cytotoxic granules exocytosis from murine NK cells via binding to an unidentified adenosine receptor (69). More recently, data support the fact that adenosine and its stable analog 2-chloroadenosine inhibit perforin- and Fas ligand-mediated cytotoxic activity as well as cytokines production (i.e., IFN-γ, macrophage inflammatory protein 1-α, TNF-α, and granulocyte-macrophage CSF) from activated NK cells. These inhibitory effects occur through the stimulation of the cyclic AMP/protein kinase A pathway following the binding of adenosine to A2A receptors on NK cells (70, 71). In this context, targeting the CD73-adenosine pathway has recently emerged as a potential clinical strategy for immunotherapy (66). *In vitro* data revealed that the inhibition of the CD39, CD73, or A2A adenosine receptor by siRNA, shRNA, or specific inhibitors resulted in a significant improvement of NK cell lytic activity against ovarian cancer cells (72). Furthermore, *in vivo* blocking of the A2A adenosine receptor enhanced NK cell activity in a perforin-dependent manner and reduced metastasis of CD73-overexpressing breast cancer cells (73).

As multiple immune competent cells express adenosine receptors, an additional level of immunomodulatory activity, via adenosine, needs to be considered. For example, several studies reported that adenosine interaction with other immune subsets impairs the cytotoxic activity, the pro-inflammatory cytokines production, and the proliferation of T cells. In addition, adenosine impairs the recruitment and the immunosuppressive activity of MDSCs in tumors, as well as the migration and the immunosuppressive function of Treg cells into the TME (74). Taken together, by sustaining the immunoregulatory activity of extracellular adenosine, all the mechanisms described above collaborate to impair the anti-tumor NK-mediated immunity.

### Nitric oxide

Accumulating evidence suggests that the exposure of cells to low oxygen levels results in a marked inhibition of NO production (75). NO is produced from l-arginine in a reaction catalyzed by the NO synthase (NOS) enzymes, in which oxygen is a required cofactor. Hypoxia has also been shown to increase arginase activity, thereby redirecting l-arginine into the urea cycle, away from the NO generation pathway (76). Siemens et al. provided evidence that hypoxia-mediated impairment of NO signaling in tumor cells contributes to tumor escape from NK immunosurveillance. They demonstrated that hypoxia-mediated shedding of MIC occurs through a mechanism involving impaired NO signaling in human prostate cancer. Such shedding can be blocked after reactivating NO signaling by the administration of NO mimetic agents (45). This work suggests that reactivation of NO could help to overcome hypoxia-driven tumor escape.

### Prostaglandin E2

Several lines of evidence suggest that the deregulation of the cyclooxygenase (COX)-2/PGE2 pathway is a key factor in tumor evasion of the immune response (77). COX enzymes catalyze the formation of prostaglandins from arachidonic acid following sequential oxidation. Interestingly, COX-2 can be overexpressed in both adenoma and carcinoma cells under hypoxia via a mechanism dependent on HIF-1α. This upregulation is associated with PGE2 overproduction and secretion in the microenvironment (78). Early studies showed that PGE2 suppresses the cytolytic activity of NK cells (79, 80) by a mechanism related to the inhibition of IFN-γ production (81, 82). Recently, Pietra et al. have shown that melanoma cells affect the function of NK cells by downregulating the surface expression of activating receptors, including NKp30, NKp44, and NKG2D. This impairment appears to be related, at least in part, to PGE2 production by melanoma cells as PGE2-specific inhibitor-restored NK cell functions (47). In addition to its direct effect on NK cells, more recent data reported that PGE2 can indirectly affect the NK cell function by promoting the establishment of an immunosuppressive microenvironment through the induction of Treg cells (83), macrophages (84), and MDSCs (27, 85) development.

### Galectins

Galectins (Gal) are proteins belonging to the lectins family that participate in the delivery of signals after binding to glycoproteins and glycolipids on the cell surface of target cells. Using a proteomic approach, Le et al. have identified Gal-1 as a novel hypoxia-regulated protein (86). They proposed that tumor aggressiveness of HNSCC is dependent on hypoxia-mediated production and the secretion of Gal-1, which in turn negatively regulates the anti-tumor immune response. Additional studies have supported the contribution of Gal-1 in creating an immunosuppressive microenvironment at the sites of tumor growth by several mechanisms (87). Thus, it has been reported that recombinant Gal-1 is able to promote the differentiation of CD4+CD25+ Treg cells *in vitro* (88). Recently, Dalotto-Moreno et al. showed that tumor-derived Gal-1 increases the abundance and/or the expansion of peripheral Treg cells *in vivo* and modulates their suppressive capacity. Conversely, attenuation of Gal-1 reduces the frequency of Treg cells within tumors, lymph nodes, and spleen and removes the immunosuppressive function of Treg cells (89). More recently, Gal-3, another member of the galectin family regulated by HIF-1α (90), was reported to exert an immunosuppressive function in the TME. Tsuboi et al. provided evidence that cell surface Gal-3 on bladder tumor cells modulates MICA-NKG2D interactions by binding MICA through poly-*N*-acetyllactosamine, thereby severely impairing the NK cell activation and degranulation (91). The effect of Gal-9 is still debated as it may regulate both positively and negatively the NK cell response depending on the activation threshold and the expression of its receptor. Gleason et al. have shown that Gal-9 binding to the immune receptor T cell Ig and mucin-containing domain-3 (Tim-3) enhances the production of IFN-γ by NK cells (92). Conversely, higher doses of Gal-9 impair the cytotoxic function of NK cells in a Tim-3 independent manner (93).

## Regulation of NK Cell-Mediated Killing by Autophagy

It has become increasingly clear that tumor cells activate key biochemical and cellular pathways under hypoxic stress that are important for tumor progression, survival, and metastasis. Several recent reports highlight autophagy as a critical process that modulates the anti-tumor immune response. Briefly, autophagy is a catabolic process in which a cell self-digests its own components. Autophagy can be activated in response to multiple stressors including hypoxia, nutrient starvation, growth factor withdrawal, and endoplasmic reticulum stress. Under stressful stimuli, autophagy activation serves as an adaptive response to provide nutrients and prevents accumulation of altered cell components (94).

**Figure 4:**
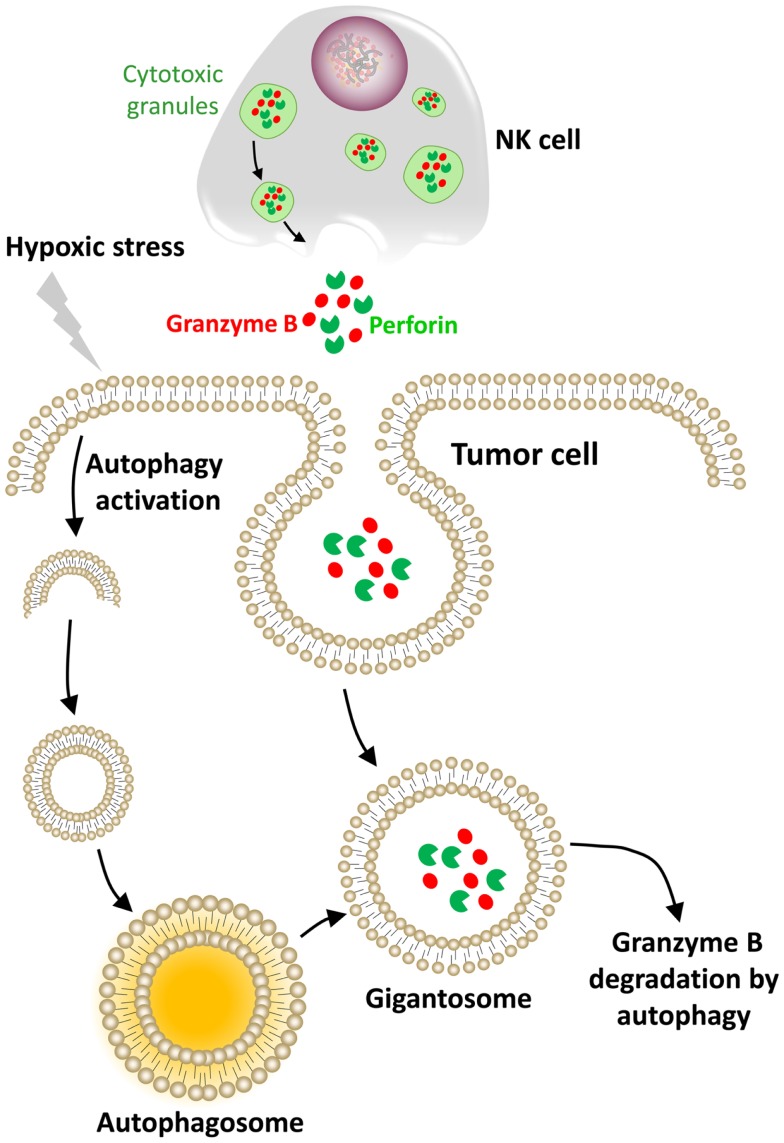
**Hypoxic stress activates autophagy in tumor cells as an intrinsic resistance mechanism to NK-mediated killing**. In our model, the cytolytic effectors perforin and granzyme B enter the target cells by endocytosis and then are found in enlarged endosomes called “gigantosomes.” In hypoxic cells, the activation of autophagy leads to the formation of autophagosomes that fuse with “gigantosomes” to form amphisomes. The fusion between amphisomes and lysosomes selectively degrades granzyme B in this compartment, making hypoxic tumor cells less sensitive to NK-mediated lysis.

To adapt to hypoxia, cells activate autophagy through both HIF-1α dependent and independent pathways, depending on the sensor activated (95). The role of autophagy in cancer immunity seems to be complex as hypoxia-induced autophagy occurs in target cells and in tumor-infiltrating immune cells. Although the role of autophagy induction in target cells is well documented, relatively little attention has been given to its role in immune cells. Therefore, understanding how autophagy modulates the tumor immune response represents a major challenge in the field of tumor immunotherapy. Recently, it has been reported that NK cells not only provide lytic signals to their target cancer cells, but also promote autophagy in the remaining un-killed target cells. Moreover, the NK-mediated autophagy induction in target cells was enhanced by provision of IL-2 and cell-cell interactions between NK cells and tumor cells. This study highlights autophagy induction in target cells as a cell mechanism of resistance to NK-mediated killing (96). More recently, we showed *in vitro* and *in vivo* that targeting autophagy under hypoxia restores NK-mediated lysis in breast cancer cells. In addition, we provided mechanistic evidence that the activation of autophagy under hypoxia led to the degradation of NK-derived granzyme B, making hypoxic tumor cells less sensitive to NK-mediated killing (Figure 4) (97).

## Tumor-Derived Extracellular Vesicles Influence NK Cell Activity

Recent advances have led to the identification of an additional mechanism used by tumor cells to escape NK cell recognition and impair the NK-mediated immune response (98). Indeed, tumor cells release vesicle-bound molecules (cytokines, NKG2D ligands, and miRNAs) targeting and inhibiting NK cell functions (99).

Exosomes are 50–150 nm membrane vesicles derived from the multi-vesicular bodies that are secreted by all cell types [reviewed in Ref. (100)]. As a consequence, exosomes are found in many biological fluids such as urine, plasma, and saliva. As their content reflects the cells from which they are derived, exosomes represent, therefore, attractive biomarkers (101). Exosomes and other types of extracellular vesicles are well-known mediators of intercellular communication and play a crucial role in the development of aggressive and metastatic tumors (102, 103).

### Cancer cell-derived exosomes

**Figure 5:**
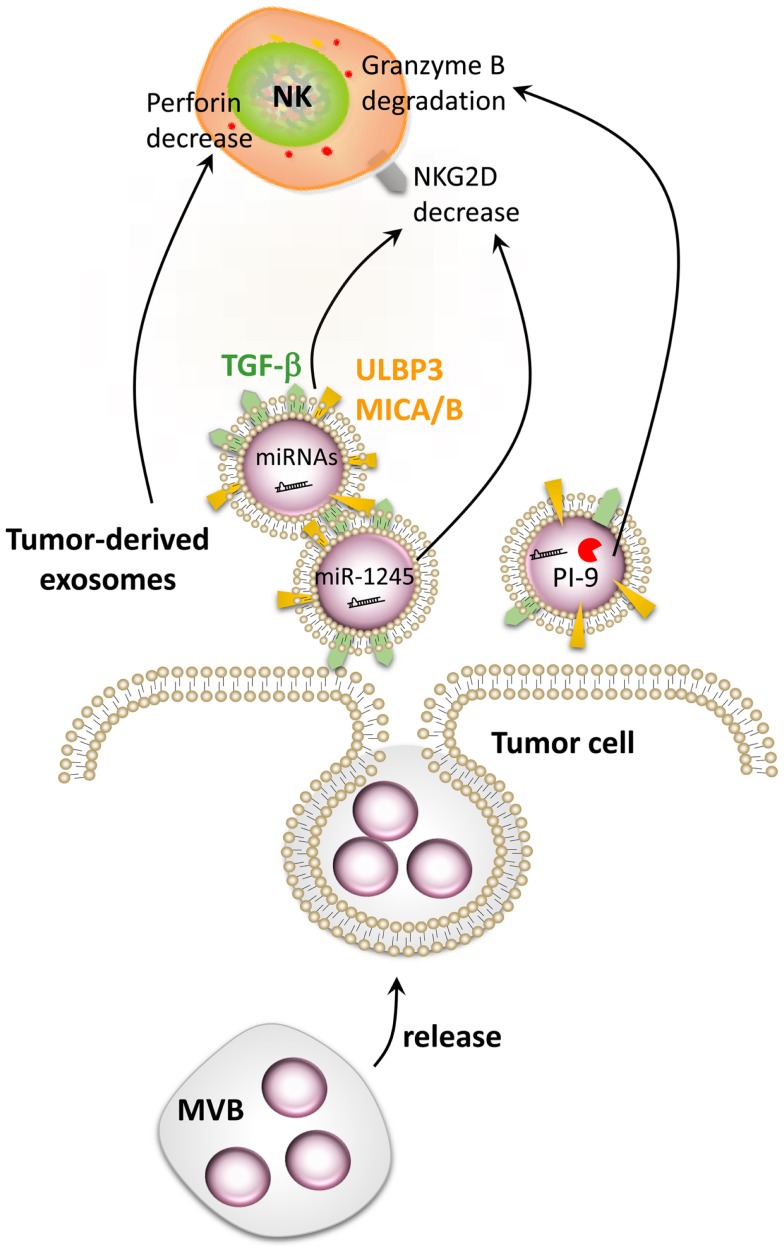
**Impairment of NK cell function by tumor-derived exosomes**. Tumor cells secrete extracellular vesicles called exosomes. Tumor-derived exosomes contain numerous factors able to modulate the function of NK cells such as MICA/B, ULBP3, TGF-β, PI-9, and different microRNAs. Exosome-derived MICA/B, ULBP3, TGF-β, and miR-1245 can decrease NKG2D on the surface of NK cells, while PI-9 degrades granzyme B. Tumor-derived exosomes can also decrease the level of perforin in NK cells by a still-unknown mechanism.

The production of NKG2D ligand-bearing exosomes has been proposed as a mechanism for tumor cell escape from immune recognition (99, 104, 105). Indeed, it has been demonstrated that, in contrast to ULPB2, released ULBP3 is included into exosomes. Remarkably, ULBP3-containing exosomes have been shown to be more potent downregulators of the NKG2D receptor than the soluble form of ULBP2 proteins released by the metalloproteinases ADAM10 and 17. Pre-incubation of NK cells with ULBP3-containing exosomes induced a dramatic reduction of NKG2D-mediated lysis of MICA-expressing cells (106). Tumor-derived exosomes (TDEs) are rapidly taken up by NK cells and remain stable for 48 h (104, 107). The transfer of TDE-bearing, membrane-anchored TGF-β, MICA, and MICB leads to the downregulation of NKG2D expression at the surface of NK cells and impairs their cytotoxic functions (Figure 5) (99, 108). However, TDEs can only weakly impair the NK cell proliferation compared with their strong negative effect on the proliferation of CD8+ T cells (109). Nevertheless, numerous studies highlighted TGF-β as a major immunosuppressive molecule for NK cells (108, 110, 111). Indeed, an elevated plasma level of TGF-β was detected in lung or colorectal cancer patients compared with healthy volunteers. This increase inversely correlated with NKG2D surface expression on NK cells in these patients (110). Recently, TGF-β was shown to block NK cell activation by repressing gene expression and antagonizing IL-15-induced proliferation (111). A striking observation was also done by Clayton et al. who identified exosomal TGF-β1 as a more potent contributor to antiproliferative effects than the soluble form (109).

Several cancer models have generated evidence supporting the important roles of TDEs. Indeed, mammary carcinoma exosomes promote tumor growth by suppressing NK cell function in mice (104). A decrease in splenic NK cell cytotoxicity was observed after *in vivo* injection of TDEs. Moreover, a reduction in the number and the percentage of NK cells was observed in the lungs 3 days after exosome injection, without a reduction in the viability of the NK cells. Interestingly, TDEs also reduced the expression of the NK pore-forming and cytolytic protein perforin (Figure 5) (104, 111), whereas the level of granzyme B was unaffected (104). A decrease in NK cell proliferation in response to IL-2 was also observed after treatment with exosomes derived from different tumor cell types (breast and melanoma) due to the inhibition of the JAK-STAT signaling. However, TDEs did not affect DC maturation but hampered their ability to stimulate the immune response (104). The granzyme B-inhibitory serpin proteinase inhibitor-9 (PI-9) has also been identified inside exosomes (112) and could also play an important role in the resistance of tumor cells to NK cells (Figure 5). Taken together, these data highlight the crucial role that TDEs may have on the tumor immunosurveillance by affecting the NK cell receptors, proliferation, and release of cytotoxic molecules, thus impairing an effective anti-cancer immune response.

Numerous studies have provided evidence that hypoxic stress may influence the composition of TDEs. Indeed, to substitute oxygen deprivation and a lack of nutrients, tumor cells induce the expression of angiogenic factors to overcome hypoxic stress through the formation of new blood vessels from existing vasculature. In addition to secreted VEGF, several chemokines (G-CSF, GM-CSF, CXCL16, and SDF-1) and exosomes were shown to be important mediators for tumor cells to overcome hypoxic stress (102). In this context, it has been reported that tumor cells under hypoxic stress secrete numerous proteins sequestered in exosomes involved in cell–cell communication, cell growth, and malignant transformation. Other studies have focused on how hypoxia-induced membrane vesicles stimulate angiogenesis in malignant and angiogenic brain tumor glioblastoma multiforme (GBM). Indeed, hypoxic cancer cells release exosomes containing tissue factor (TF) acting on surrounding endothelial cells in a paracrine manner, leading to the activation of a protease-activated receptor 2 (PAR2)-ERK signaling pathway (113). PAR2 has been recently identified as a regulator of the innate immune response and a mediator of cell proliferation and migration. Also called thromboplastin, TF forms a complex with the tissue protease factor VIIa and is necessary for the initiation of thrombin formation. Because hypoxic tumors are often characterized by endothelial cell hyperplasia and hypercoagulation, the combined presence of newly generated fibrin and activated platelets has been shown to protect the tumor from NK cells and immune surveillance (114). Further findings obtained with GBM cells indicates that hypoxic conditions stimulated tumor cells to generate exosomes containing proteins that reflect the hypoxic status of the tumor cells. These findings support the hypothesis that the microenvironment significantly impacts the TDE composition. The enrichment in exosomes of specific hypoxia-related RNAs and proteins (cytokines, growth factors, and MMP) could indeed be associated with a poor patient prognosis. In addition, hypoxic TDEs mediated a strong paracrine stimulation of angiogenesis and activation of cancer cells, leading to an acceleration of tumor growth in a mouse xenograft model (115). TDEs systematically contain several members of the ADAM family, mostly ADAM10 (107), which is able to shed NKG2D ligands from the cell membrane (116). Finally, besides stimulating the production of exosomes with a specific content, hypoxia has also been shown to enhance exosome release by cancer cells (92).

Besides solid tumors, circulating tumor cells, such as leukemic cells, escape NK surveillance at a systematic level in blood. It is important to note that leukemic cells are constantly recirculating in the bone marrow, where the environment is maintained in constant hypoxia (117). Recent studies have shed light on mechanisms of tumor cell escape from NK-mediated killing that could be used as new therapeutic approaches. These mechanisms include the shedding of soluble (BAG6, and MICA) or exosome-derived inhibitory molecules (TGF-β) in various malignancies such as acute myeloid leukemia (118), chronic lymphocytic leukemia (119), and Hodgkin’s lymphoma (119).

### Secreted microRNAs

As described above, under hypoxic conditions, most cell types undergo important metabolic changes orchestrated by members of the HIF transcription factor family. It is well documented that HIF-1α is a potent inducer of miR-210 (120), which has been described to be released by tumor cells (121, 122). It has been shown that miR-210 released by leukemic and metastatic cancer cells may be transported by exosomes and enter endothelial cells (121, 122). In the recipient cells, miR-210 is able to induce angiogenesis and promote tumor growth. These data highlight the role of exosomal miR-210 in the shaping of the TME and the potential action on various cell types present at the tumor site. Although the data available are limited, we believe that exogenous miRNAs can impair the anti-tumor function of immune cells (Figure 5). In line with this concept, it has been shown that the TGF-β1-induced miR-1245 downregulated the NKG2D receptor on NK cells and impaired NKG2D-mediated functions (123). The influence of exogenous miRNAs on NK cells is currently unknown but understanding this new regulatory mechanism may help to improve the outcome of NK-based immunotherapy.

## Conclusion

Recent developments in cancer immunotherapies have now begun to explore the use of NK cells (15, 124). Particularly, strategies designed to improve NK-mediated killing using tumor-specific mAbs have shown promising results in preclinical and some clinical settings (125). This review has summarized the different mechanisms involved in the impairment of NK-mediated tumor killing and highlighted that the majority of these mechanisms likely evolve within the TME. In this regard, it should be emphasized that the composition and characteristics of the TME are important in determining the anti-tumor immune response. For example, different subsets of the immune system, including NK cells, DCs, and effector T cells, are capable of driving potent anti-tumor responses. However, the ability of tumor cells to exploit other cells present in the TME is now widely regarded as a critical factor that switches the immune response from a tumor-destructive profile to a tumor-promoting profile. Such a microenvironment may also favor the development of immunosuppressive populations of immune cells, such as MDSCs, TAMs, and Treg cells.

Despite recent advances in cancer immunotherapy, the therapeutic outcome was often disappointing in many clinical protocols. Given the important immunomodulatory effects of the TME, it stands to reason that it may represent a therapeutic target that can be manipulated to improve the anti-tumor immune response. Thus, the first clinical interventions that aim to target the microenvironment to enhance tumor immunity are under active evaluation.

Overall, investigations oriented toward the identification of novel therapeutic strategies, aiming to improve the anti-tumor immunotherapy, should pay closer attention to the TME to awake or reawake immune cells and/or to redirect such a microenvironment from a pro-tumor to an anti-tumor state. Given its central role in tumor progression and resistance to therapy, the hypoxic TME should be considered as a new critical therapeutic target in oncology. We believe that a better characterization of the TME can provide important prognostic and predictive values independent of the tumor phenotype.

## Conflict of Interest Statement

The authors declare that the research was conducted in the absence of any commercial or financial relationships that could be construed as a potential conflict of interest.
